# Elevated hemoglobin is independently associated with enlarged perivascular spaces in the central semiovale

**DOI:** 10.1038/s41598-021-82327-9

**Published:** 2021-02-02

**Authors:** Yingchao Huo, Siyuan Huang, Rui Li, Xue Gong, Wenyu Zhang, Rongrong Zhang, Xinyue Qin

**Affiliations:** 1 Department of Neurology, The First Affiliated Hospital of Chongqing Medical University, Chongqing, 400016 People’s Republic of China; 2 Division of Life Sciences and Medicine, Department of Neurology, The First Affiliated Hospital of USTC, University of Science and Technology of China, Hefei, 230001 Anhui People’s Republic of China; 3 Department of Neurology, Panzhihua Municipal Central Hospital, Panzhihua, 617000 Sichuan People’s Republic of China

**Keywords:** Diseases, Neurology

## Abstract

Enlarged perivascular spaces (EPVS) are widely considered as a feature of cerebral small vessel diseases (SVD), but its underlying pathology is still under active investigation. The aim of this study was to explore the association between hemoglobin level and the severity of EPVS. Consecutive patients with acute ischemic stroke who underwent baseline MRI scan and hemoglobin testing were evaluated. EPVS in basal ganglia (BG) and central semiovale (CS) were rated with a validated 4-point semiquantitative scale (0 = none; 1 = 1–10; 2 = 11–20; 3 = 21–40; and 4 ≥ 40). Bivariate logistic regression models were used to identify the associations of hemoglobin with predefined high-degree (score > 1) CS-EPVS and BG-EPVS. Multinomial logistic regression models were used to analyze the associations between hemoglobin and CS-/BG-EPVS predominance patterns. A total of 401 patients were included in the final analysis, 94 patients (23.4%) had a high degree of CS-EPVS and 45 patients (11.2%) had a high degree of BG-EPVS. Compared with tertile 1 of hemoglobin, tertile 3 of hemoglobin was independently associated with high degree of CS-EPVS after adjusting for other features of SVD (odds ratio [OR] 2.399, 95% confidence interval [CI] 1.315–4.379, P = 0.004) and potential confounding factors (OR 2.611, 95% CI 1.346–5.066, P = 0.005). In multinomial logistic regression models, compared with tertile 1 of hemoglobin, tertile 2 (OR 2.463, 95% CI 1.195–5.075, P = 0.015) and tertile 3 (OR 2.625, 95% CI 1.102–6.251, P = 0.029) of hemoglobin were associated with higher odds of BG-EPVS = CS-EPVS pattern, and tertile 3 of hemoglobin (OR 2.576, 95% CI 1.004–6.608, P = 0.049) was associated with higher odds of BG-EPVS < CS-EPVS pattern. Elevated hemoglobin level was independently associated with high degree of CS-EPVS and higher odds of CS-EPVS predominance pattern, but not with BG-EPVS, which support that the topography of EPVS is characteristic. However, the pathogenesis linking hemoglobin and CS-EPVS is unclear and still needs further investigation.

## Introduction

Perivascular spaces (also known as Virchow–Robin spaces) are tiny cavities around small perforating arteries that serving as an important drainage system for interstitial fluid (ISF) and solutes in the brain^1^. When enlarged, they become detectable by magnetic resonance imaging (MRI), especially in the basal ganglia (BG) and central semiovale (CS)^2^. Although it is normal to have a few visible enlarged perivascular spaces (EPVS) on MRI^3^, EPVS are widely recognized as a feature of cerebral small vessel diseases (SVD)^4,5^. Several studies have found that increased burden of EPVS are associated with hypertension^6,7^, intracranial atherosclerosis^8^, cognitive impairment^9^, dementia^10^, sleep disorder^11^ and depression^12^. However, underlying pathologies of EPVS are still unclear and under active investigation.

Accumulating evidence suggests that the topography of EPVS is characteristic and EPVS in BG or CS might have different origins^7,13,14^. EPVS in BG are associated with other features of SVD^4,6,7,15^ and have been demonstrated as a marker of hypertensive arteriopathy^7,16^. However, EPVS in CS have a higher prevalence in patients with cerebral amyloid angiopathy (CAA) and are strongly associated with lobar cerebral microbleeds (CMB) and superficial siderosis^7,13,14,17^. Therefore, it has been hypothesized that in contrast to BG-EPVS, CS-EPVS may be a neuroimaging marker of CAA by representing ISF elimination dysfunction due to vascular amyloid deposition^14,18^.

Previous study suggested that hemoglobin, the iron-containing oxygen transport metalloprotein in the red blood cells, could reflect the level of oxygen consumption and might relate to blood viscosity which is associated with stroke risk when increased. What’s more, hemoglobin has been demonstrated associated with prognosis after ischemic stroke^19^. Besides, hemoglobin was associated with the severity of CAA and might be involved in the pathophysiology of CAA^20^. However, whether EPVS, one of the main features of SVD, is associated with hemoglobin level remained unknown. To the best of our knowledge, the data for relationship between hemoglobin level and EPVS is scarce. Investigation of the relationship between hemoglobin level and EPVS is of great significance to explore the pathogenetic mechanism of EPVS. Thus, in the present study, we aimed to explore the association between hemoglobin level and the severity of EPVS.

## Methods

### Study population

For this retrospective analysis, we used prospectively collected data from a database involving consecutive patients admitted at the Department of Neurology of First Affiliated Hospital of Chongqing Medical University between April 2017 and January 2018 with acute ischemic stroke^21^. Acute ischemic stroke was diagnosed if there were new focal neurological deficits explained by relevant lesions detected on diffusion-weighted imaging (DWI) or computed tomography (CT). The inclusion criteria include: (1) had complete cerebral MRI examination data including T1-weighted, T2-weighted, fluid attenuated inversion recovery (FLAIR) and DWI sequences; and (2) undergone blood sample collection for hemoglobin testing within 12 h of admission. Patients were excluded if they: (1) had contraindication to MRI or with poor quality of MRI; (2) had severe acute ischemic stroke that lead to difficulty in EPVS assessment; (3) with inadequate clinical information or laboratory data; (4) with other severe neurological disease including dementia, Parkinson disease, severe traumatic or toxic or infectious brain injury, and brain tumor; (5) with serious diseases including acute myocardial infarction, severe heart failure, severe infections, severe respiratory failure, severe nephrosis or liver disease, or cancer; and (6) refused to participate in the study. The study was approved by the Ethics Committee of The First Affiliated Hospital of Chongqing Medical University and all participants or their next-of-kin gave written, informed consent. The study protocol was performed in accordance with the Declaration of Helsinki.

### Data collection

Clinical data that included demographic data and medical history data were collected on admission. Demographic data included age, sex and body mass index (BMI). Medical history data comprised hypertension, diabetes mellitus, hypercholesterolemia, current smoking, alcohol consumption and coronary heart disease. The definition of these factors was confirmed as previously described^21^.

Fasting blood sample was collected from each patient after admission and was sent to the clinical laboratory of First Affiliated Hospital of Chongqing Medical University for blood routine, blood lipid, fasting plasma glucose (FPG), glycosylated hemoglobin A1c (HbA1c), and homocysteine (Hcy) examination. Blood routine indices including white blood cell (WBC) count, platelet count, hemoglobin, hematocrit, mean corpuscular volume (MCV) and red blood cell distribution width (RDW) were tested using an automated biochemical analyzer. Blood lipid indices including total cholesterol (TC), triglyceride (TG), low-density lipoprotein cholesterol (LDL-c), high-density lipoprotein cholesterol (HDL-c), apolipoprotein A-I (Apo A-I), and apolipoprotein B (Apo B).

### Imaging

Brain MRI was performed on the day of admission by a 3.0T magnet scanner (GE Medical Systems, Waukesha, WI, USA). Sequences included T1-weighted, T2-weighted, FLAIR and DWI sequences. All MRI scans were evaluated by an neurologist specialized in stroke (Huo, YC with 6 years experiences) who was blind to clinical information.

EPVS were defined as sharp delineated lesions with signal intensity similar to cerebrospinal fluid on all sequences and (if visible) without a hyperintense rim on FLAIR sequence to distinguish from lacunes^22^. They appeared round or linear-shaped with diameters < 3 mm. We counted the number of EPVS in the CS and BG separately and rated with a validated 4-point semiquantitative scale (0 = no EPVS; 1 = 1–10 EPVS; 2 = 11–20 EPVS; 3 = 21–40 EPVS; and 4 ≥ 40 EPVS)^23^. At both regions, the number refers to the highest EPVS in the most affected hemisphere. Definitions of lacunes, periventricular white matter hyperintensities (PVWMH) and deep white matter hyperintensities (DWMH) are provided in the Supplementary material. To ensure the reliability of data and results, another experienced neurologist Li who is specialized in neuroimaging with 14 years experiences was invited for limited intrarater reliability testing (50 scans). Consensus analysis was made by joint discussion in cases of discrepancies. The intrarater κ values showed a good reliability for the presence of BG-EPVS (0.82), CS-EPVS (0.75), PVWMH (0.89), DWMH (0.85) and lacunes (0.81).

### Statistical analysis

The degrees of BG-EPVS and CS-EPVS were dichotomized as high (score > 1) or low (score ≤ 1)^4,8^. The burden of BG-EPVS and CS-EPVS were also compared in each patient and then divided into 3 predominance patterns: higher degree of CS-EPVS (i.e., BG-EPVS < CS-EPVS), equal degree in the 2 regions (i.e., CS-EPVS = BG-EPVS), and higher degree of BG-EPVS (i.e., BG-EPVS > CS-EPVS)^14^. Besides, hemoglobin was categorized into tertiles and also considered as a continuous variable to evaluate the association with EPVS. In univariate analyses, characteristics of patients were compared using independent samples t test, one-way analysis of variance, Mann–Whitney U test or Kruskal–Wallis H test for continuous variables and Pearson’s chi square test or Fisher’s exact test for categorical variables as appropriate.

We assessed the associations between hemoglobin and BG-/CS-EPVS by binary logistic regression. The associations were firstly analyzed adjusting for age and sex, and then adjusting for other features of SVD as well as additional confounding factors. We subsequently evaluated associations between hemoglobin and BG-/CS-EPVS predominance patterns in unadjusted and adjusted (age and sex) multinomial logistic regression models. The base outcome for these analyses was the BG-EPVS predominance pattern (i.e., BG-EPVS > CS-EPVS). The statistical analyses were done with SPSS software (IBM Corp., Armonk, NY, USA, version 19.0) and STATA software (STATA Corp., College Station, Texas, USA, version 12.0). Significance level was considered at P < 0.05 (2 tailed).

## Results

### Characteristics of EPVS in BG and CS

**Figure 1:**
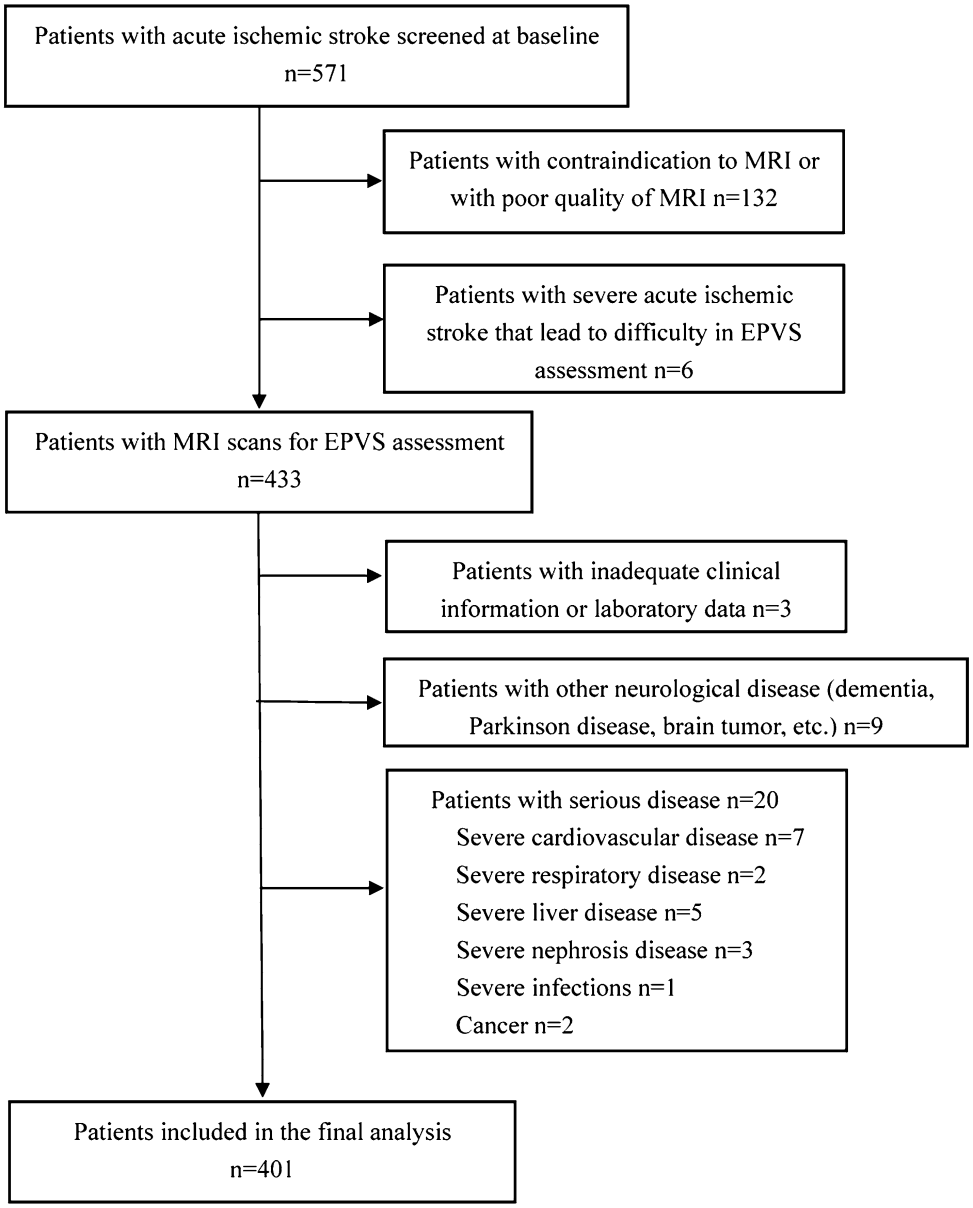
Flow chart of recruitment.

**Figure 2:**
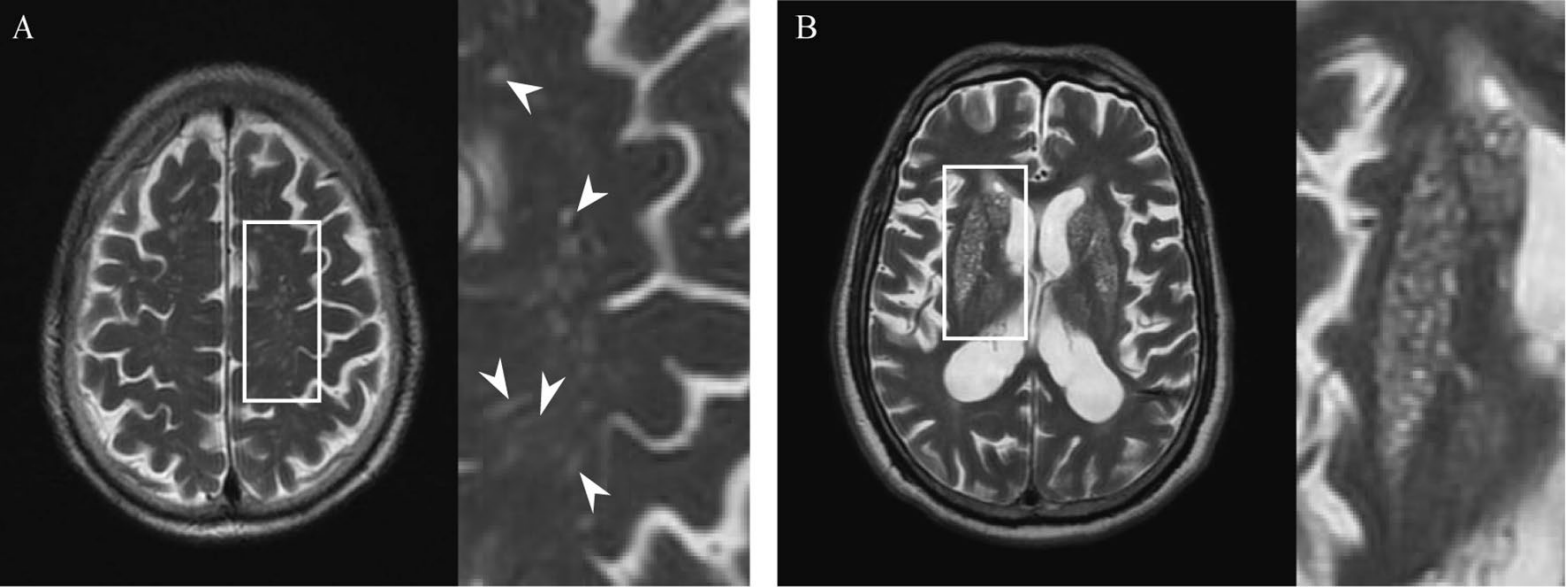
Representative examples of high degree of CS-EPVS (**A**) and BG-EPVS (**B**). Arrowheads point to individual EPVS. *EPVS* enlarged perivascular spaces, *BG* basal ganglia, *CS* centrum semiovale.

Our final analysis included 401 patients with acute ischemic stroke (Fig. 1). Average age was 66 years (age range 28–94), with 273 males (68.1%) and 128 females (31.9%). As to degree of EPVS, 94 patients (23.4%) had a high degree of EPVS in the CS and 45 patients (11.2%) had a high degree of EPVS in the BG. Representative examples are illustrated in Fig. 2. Clinical and imaging characteristics of patients with high and low EPVS degree in BG and CS are presented in Table 1.

**Table 1:** Comparison of characteristics between patients with high and low EPVS degree in BG and CS.

	Degree of CS-EPVS			Degree of BG-EPVS		
	Low (n = 307)	High (n = 94)	*P* value	Low (n = 356)	High (n = 45)	*P* value
Age, years, median (IQR)	67 (59–76)	64 (55–73)	0.213	65 (57–74)	75 (67–81)	0.000
Gender, male, n (%)	198 (64.5)	75 (79.8)	0.005	242 (68.0)	31 (68.9)	0.902
BMI, median (IQR)	23.79 (21.48–25.71)	24.51 (21.97–26.28)	0.323	24.04 (21.68–25.71)	22.86 (20.13–25.51)	0.088
Hypertension, n (%)	209 (68.1)	71 (75.5)	0.168	242 (67.9)	38 (84.4)	0.023
Diabetes mellitus, n (%)	128 (41.7)	46 (48.9)	0.215	157 (44.1)	17 (37.8)	0.420
Hypercholesterolemia, n (%)	115 (37.5)	49 (52.1)	0.011	145 (40.7)	19 (42.2)	0.848
Coronary heart disease, n (%)	52 (16.9)	16 (17.0)	0.985	61 (17.1)	7 (15.6)	0.790
Current smoking, n (%)	126 (41.0)	44 (46.8)	0.322	156 (43.8)	14 (31.1)	0.104
Alcohol consumption, n (%)	77 (25.1)	21 (22.3)	0.588	89 (25.0)	9 (20.0)	0.462
Cerebral atrophy, n (%)	67 (21.8)	23 (24.5)	0.575	54 (15.2)	36 (80.0)	0.000
TC, mmol/L, median (IQR)	4.36 (3.75–5.08)	4.75 (4.05–5.22)	0.065	4.43 (3.79–5.16)	4.53 (3.74–5.17)	0.913
TG, mmol/L, median (IQR)	1.38 (1.01–1.97)	1.51 (1.20–2.21)	0.028	1.27 (0.91–1.46)	1.46 (1.04–2.11)	0.032
HDL-c, mmol/L, median (IQR)	1.10 (0.92–1.34)	1.05 (0.83–1.33)	0.119	1.09 (0.89–1.33)	1.09 (0.98–1.34)	0.338
LDL-c, mmol/L, median (IQR)	2.83 (2.26–3.40)	3.12 (2.34–3.55)	0.184	2.88 (2.29–3.42)	2.95 (2.38–3.59)	0.689
Apo A-I, median (IQR)	1.22 (1.07–1.38)	1.19 (1.09–1.37)	0.606	1.21 (1.07–1.38)	1.21 (1.09–1.36)	0.886
Apo B, median (IQR)	0.94 (0.77–1.14)	1.02 (0.86–1.17)	0.065	0.97 (0.80–1.14)	0.95 (0.74–1.16)	0.721
HbA1C, median (IQR)	6.05 (5.7–7.425)	6.15 (5.8–7.9)	0.316	6.1 (5.7–7.55)	6.05 (5.65–6.7)	0.347
Fasting plasma glucose, mmol/L, median (IQR)	5.9 (5.1–7.5)	5.7 (5–8.65)	0.875	5.9 (5.1–8.0)	5.5 (5.03–6.75)	0.091
Hcy, median (IQR)	12.4 (10.3–15.5)	12.7 (10.7–15.3)	0.765	12.3 (10.3–15.2)	14.5 (10.7–17.7)	0.032
WBC, 10^9^/L, median (IQR)	7.05 (5.98–8.85)	6.89 (5.56–8.53)	0.345	7.03 (5.90–8.85)	6.99 (6.07–8.20)	0.773
Platelet, 10^9^/L, median (IQR)	184 (150–229)	196 (154–222)	0.543	187 (148–228)	198 (160–222)	0.339
MCV, fL, median (IQR)	91.6 (88.5–93.9)	91 (87.5–94.7)	0.245	91.5 (88.2–93.8)	91.4 (88.3–95.7)	0.661
RDW, %, median (IQR)	13.3 (12.8–14.0)	13.3 (12.8–14.1)	0.906	13.3 (12.7–14.0)	13.5 (13.0–14.4)	0.159
Onset to admission time, hour, median (IQR)	16.5 (5.0–40.5)	18.3 (7.0–40.4)	0.211	16.5 (5.0–40.9)	18.5 (7.0–34.0)	0.580
**Stroke subtype, n (%)**			0.105			0.694
Large artery atherosclerosis	97 (31.6)	32 (34.0)		112 (31.5)	17 (37.8)	
Cardioembolism	62 (20.2)	11 (11.7)		66 (18.5)	7 (15.6)	
Small vessel occlusion	99 (32.2)	41 (43.6)		124 (34.8)	16 (35.6)	
Undetermined	11 (3.6)	1 (1.1)		12 (3.4)	0 (0)	
Other determined	38 (12.4)	9 (9.6)		42 (11.8)	5 (11.1)	
**Location of stroke, n (%)**			0.411			0.338
Anterior cycle	198 (64.5)	54 (57.4)		228 (64.0)	24 (53.3)	
Posterior cycle	89 (29.0)	34 (36.2)		105 (29.5)	18 (40.0)	
Both anterior and posterior cycle	20 (6.5)	6 (6.4)		23 (6.5)	3 (6.7)	
Lacunes, median (IQR)	0 (0–2)	0 (0–2)	0.629	0 (0–1)	1 (0–2)	0.006
PVWMH, median (IQR)	2 (1–2)	2 (1–2)	0.693	1 (1–2)	2 (2–3)	0.000
DWMH, median (IQR)	1 (0–2)	1 (1–1)	0.501	1 (0–1)	1 (1–2)	0.000

*EPVS* enlarged perivascular spaces, *BG* basal ganglia, *CS* centrum semiovale, *IQR* interquartile range, *BMI* body mass index, *SD* standard deviations, *TC* total cholesterol, *TG* triglyceride, *LDL-c* low-density lipoprotein cholesterol, *HDL-c* high-density lipoprotein cholesterol; *Apo A-I* apolipoprotein A-I, *Apo B* apolipoprotein B, *HbA1c* glycosylated hemoglobin A1c, *FPG* fasting plasma glucose, *Hcy* homocysteine, *WBC* white blood cell, *MCV* mean corpuscular volume, *RDW* red blood cell distribution width, *PVWMH* periventricular white matter hyperintensities, *DWMH* deep white matter hyperintensities.

The prevalence of male, hypercholesterolemia and the level of TG were higher among patients with high degree of CS-EPVS vs low degree of CS-EPVS (P < 0.05). High degree of BG-EPVS was significantly associated with older age, higher prevalence of hypertension and cerebral atrophy, higher level of TG and Hcy, and higher levels of other SVD features (lacune, PVWMH, and DWMH) in univariable analyses (P < 0.05). Further analysis of the severity of SVD features found that the proportion of high BG-EPVS degree was also significantly increased with increasing severity of lacune, PVWMH, and DWMH (P < 0.05) (Supplementary Table S1).

### Characteristics of hemoglobin

Characteristics of study population grouped by tertiles of hemoglobin are presented in Table 2. Patients with higher hemoglobin tertiles were younger, tended to be more male, with higher level of BMI, TG, LDL-c, Apo B, HbA1C, CS-EPVS and total EPVS, and had higher prevalence of hypercholesterolemia, current smoking and alcohol consumption, but with lower level of HDL-c (P < 0.05). As to other feathers of SVD, the level of PVWMH, DWMH also showed significant differences among tertiles of hemoglobin (P < 0.05).

**Table 2:** Characteristics of study population grouped by tertiles of hemoglobin.

	Tertiles of hemoglobin			*P* value
	Tertile 1 (< 132) (n = 132)	Tertile 2 (132–146) (n = 134)	Tertile 3 (> 146) (n = 135)	
Age, years, median (IQR)	69 (61–80)	69 (63–76)	62 (53–69)	0.000
Gender, male, n (%)	58 (43.9)	93 (69.4)	122 (90.4)	0.000
BMI, median (IQR)	23.41 (20.90–25.12)	24.02 (21.78–25.71)	24.49 (22.04–26.35)	0.007
Hypertension, n (%)	87 (65.9)	97 (72.4)	92 (68.1)	0.510
Diabetes mellitus, n (%)	50 (37.9)	64 (47.8)	60 (44.4)	0.255
Hypercholesterolemia, n (%)	42 (31.8)	58 (43.3)	64 (47.4)	0.028
Coronary heart disease, n (%)	29 (22.0)	24 (17.9)	15 (11.1)	0.057
Current smoking, n (%)	32 (24.2)	60 (44.8)	78 (57.8)	0.000
Alcohol consumption, n (%)	17 (12.9)	32 (23.9)	49 (36.3)	0.000
TC, mmol/L, median (IQR)	4.26 (3.62–5.05)	4.41 (3.78–5.06)	4.58 (3.90–5.33)	0.077
TG, mmol/L, median (IQR)	1.25 (0.91–1.70)	1.41 (1.04–1.87)	1.71 (1.20–2.37)	0.000
HDL-c, mmol/L, median (IQR)	1.13 (0.97–1.38)	1.11 (0.92–1.36)	1.02 (0.85–1.24)	0.005
LDL-c, mmol/L, median (IQR)	2.73 (2.05–3.27)	2.92 (2.25–3.40)	3.08 (2.43–3.62)	0.013
Apo A-I, median (IQR)	1.23 ( (1.07–1.40)	1.21 (1.09–1.41)	1.19 (1.07–1.33)	0.337
Apo B, median (IQR)	0.90 (0.74–1.09)	0.98 (0.79–1.13)	1.03 (0.86–1.19)	0.001
HbA1C, median (IQR)	5.9 (5.5–7.1)	6.1 (5.8–7.4)	6.15 (5.8–8.6)	0.018
FPG, mmol/L, median (IQR)	5.7 (5.05–7.3)	5.85 (5.00–7.4)	6.1 (5.1–9.45)	0.214
Hcy, median (IQR)	12.15 (9.13–14.78)	12.50 (10.70–16.80)	12.70 (10.50–15.28)	0.144
Lacunes, median (IQR)	0 (0–1.75)	0 (0–2)	0 (0–1)	0.862
PVWMH, median (IQR)	2 (1–2)	2 (1–2)	1 (1–2)	0.021
DWMH, median (IQR)	1 (0–1.75)	1 (1–2)	1 (0–1)	0.044
BG-EPVS, median (IQR)	1 (1–1)	1 (1–1)	1 (1–1)	0.492
CS-EPVS, median (IQR)	1 (1–1)	1 (1–1)	1 (1–2)	0.000
Total EPVS, median (IQR)	2 (1–2)	2 (2–3)	2 (2–3)	0.006

*IQR* interquartile range, *BMI* body mass index, *TC* total cholesterol, *TG* triglyceride, *LDL-c* low-density lipoprotein cholesterol, *HDL-c* high-density lipoprotein cholesterol; *Apo A-I* apolipoprotein A-I, *Apo B* apolipoprotein B, *HbA1c* glycosylated hemoglobin A1c, *FPG* fasting plasma glucose, *Hcy* homocysteine, *PVWMH* periventricular white matter hyperintensities, *DWMH* deep white matter hyperintensities, *EPVS* enlarged perivascular spaces, *BG* basal ganglia, *CS* centrum semiovale.

### Hemoglobin and EPVS in CS and BG

**Figure 3:**
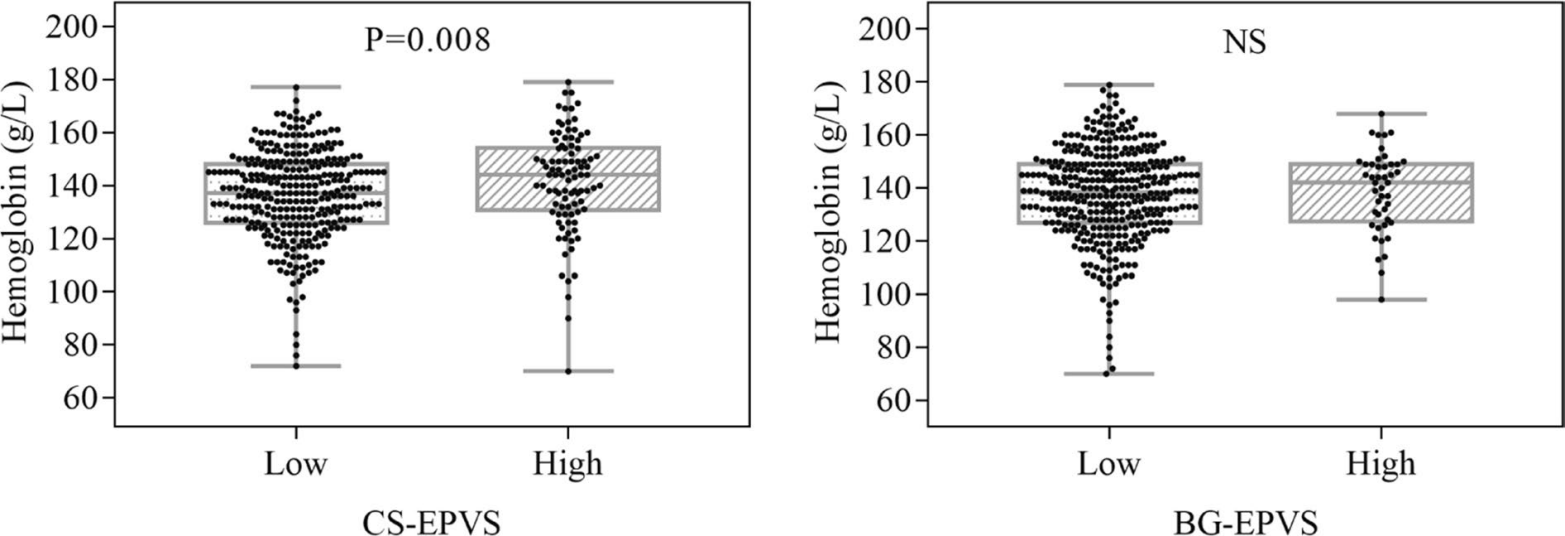
Comparison of the level of hemoglobin between patients with high and low EPVS degree in BG and CS. Box plots for hemoglobin according to the degree of EPVS in CS and BG. Bars show median values, boxes are the 25th and 75th percentiles (interquartile range, IQR), and the ends of the vertical lines are the lowest and the highest value. *NS* not significant, *EPVS* enlarged perivascular spaces, *BG* basal ganglia, *CS* centrum semiovale.

Comparison of the level of hemoglobin between high and low EPVS degree in BG and CS are presented in Fig. 3. The level of hemoglobin in patients with high degree of CS-EPVS was higher than that in patients with low degree of CS-EPVS [144 (131–154) vs 137 (126–148), P = 0.008]. Whereas, the level of hemoglobin showed no difference among patients with high vs low degree of BG-EPVS [142 (128–149) vs 139 (127–149), P = 0.589].

The prevalence of high degree of CS-EPVS and BG-EPVS in tertiles (tertile 1-tertile 3) of hemoglobin were 18.2% and 10.6%, 20.1% and 10.4%, 31.9% and 12.6%, respectively. With increasing tertiles of hemoglobin, the prevalence of high degree of CS-EPVS increased significantly (P = 0.017), but the prevalence of high degree of BG-EPVS showed no differences (P > 0.05) (Supplementary Fig. S1). As to other feathers of SVD, the distribution of PVWMH (P_trend_ = 0.088) and DWMH (P_trend_ = 0.066) severity showed no significant differences between tertiles (tertile 1–tertile 3) of hemoglobin (Supplementary Fig. S2).

**Figure 4:**
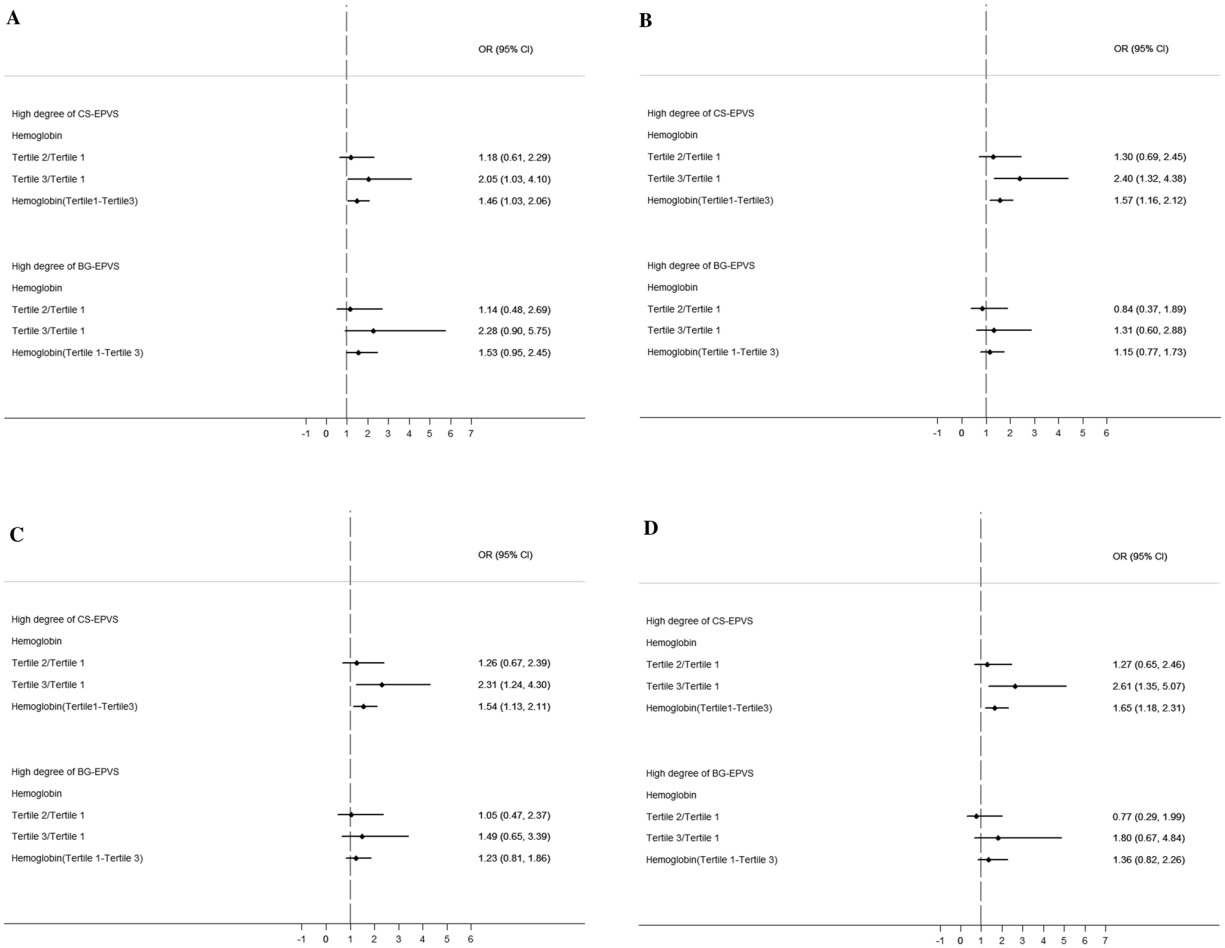
Associations between hemoglobin and high EPVS degree in BG and CS. Binary logistic regression was used to analyze these associations. (**A**) Adjusted for age and sex; (**B**) adjusted for lacune, PVWMH and DWMH; (**C**) adjusted for TC, TG and Apo B; (**D**) adjusted for BMI, hypertension, diabetes, hypercholesterolemia, coronary heart disease, current smoking, alcohol consumption, cerebral atrophy. *BG* basal ganglia, *CS* centrum semiovale, *EPVS* enlarged perivascular spaces, *PVWMH* periventricular white matter hyperintensities, *DWMH* deep white matter hyperintensities, *BMI* body mass index, *TC* total cholesterol, *TG* triglyceride, *Apo B* apolipoprotein B, *OR* odds ratio, *CI* confidence interval.

We assessed the associations between hemoglobin and high EPVS degree in BG and CS with logistic regression models, the results are presented in Fig. 4. Compared with tertile 1 of hemoglobin, tertile 3 of hemoglobin was independently associated with high degree of CS-EPVS after adjusted for age and sex (OR 2.054, 95% CI 1.030–4.099, P = 0.041; Fig. 4A). And the association remained significantly after adjusted for other features of SVD (OR 2.399, 95% CI 1.315–4.379, P = 0.004; Fig. 4B) and other potential confounding variables (OR 2.308, 95% CI 1.239–4.30, P = 0.008, Fig. 4C; OR 2.611, 95% CI 1.346–5.066, P = 0.005; Fig. 4D). Besides, hemoglobin was independently associated with high degree of CS-EPVS in all models when treated the tertiles of hemoglobin as a continuous variable (Fig. 4A, OR 1.458, 95% CI 1.030–2.064, P = 0.033; B, OR 1.569, 95% CI 1.159–2.124, P = 0.004; C, OR 1.541, 95% CI 1.126–2.110, P = 0.007; D, OR 1.653, 95% CI 1.184–2.309, P = 0.003). However, hemoglobin was not associated with high degree of BG-EPVS in all models (Fig. 4).

### Hemoglobin and EPVS degree predominance patterns

**Figure 5:**
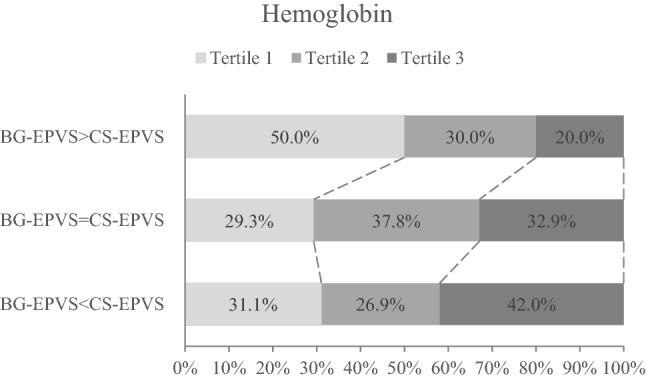
Distribution of the tertiles (tertile 1–tertile 3) of hemoglobin in patients grouped by 3 EPVS predominance patterns. The percentage of each tertile is shown in each cell. The proportion of hemoglobin tertiles (tertile 1–tertile 3) in patterns from BG-EPVS predominance to CS-EPVS predominance showed significant difference (P = 0.004), and the proportion of Tertile 3 from BG-EPVS predominance pattern to CS-EPVS predominance pattern increased significantly (P = 0.012). *BG* basal ganglia, *CS* centrum semiovale, *EPVS* enlarged perivascular spaces.

Approximately 15.0% of the patients had a BG-EPVS predominance pattern (BG-EPVS burden > CS-EPVS burden), 55.4% of the patients had the same burden of CS-EPVS and BG-EPVS and 29.7% of the patients had a CS-EPVS predominance pattern (BG-EPVS burden < CS-EPVS burden). As to tertiles of hemoglobin, the proportion of tertile 1, tertile 2 and tertile 3 in patterns from BG-EPVS predominance to CS-EPVS predominance were 50.0%, 29.3% and 31.1%, 30.0%, 37.8% and 26.9%, and 20.0%, 32.9% and 42.0%, respectively. The proportion of hemoglobin tertiles (tertile 1–tertile 3) in patterns from BG-EPVS predominance to CS-EPVS predominance showed significant difference (P = 0.004), and the proportion of tertile 3 increased significantly from BG-EPVS predominance pattern to CS-EPVS predominance pattern (P = 0.012, Fig. 5).

In multinomial logistic regression, compared with tertile 1 of hemoglobin, tertile 2 of hemoglobin (model 1, OR 2.154, 95% CI 1.104–4.201, P = 0.024; model 2, OR 2.463, 95% CI 1.195–5.075, P = 0.015) and tertile 3 of hemoglobin (model 1, OR 2.808, 95% CI 1.329–5.934, P = 0.007; model 2, OR 2.625, 95% CI 1.102–6.251, P = 0.029) were associated with higher odds of BG-EPVS = CS-EPVS pattern; tertile 3 of hemoglobin (model 1, OR 3.378, 95% CI 1.529–7.466, P = 0.003; model 2, OR 2.576, 95% CI 1.004–6.608, P = 0.049) was associated with higher odds of BG-EPVS < CS-EPVS pattern (reference group: BG-EPVS > CS-EPVS for all comparisons) (Table 3). Besides, hemoglobin was associated with higher odds of BG-EPVS = CS-EPVS pattern (model 1, OR 1.690, 95% CI 1.170–2.441, P = 0.005; model 2, OR 1.679, 95% CI 1.085–2.598, P = 0.020) and BG-EPVS < CS-EPVS pattern (model 1, OR 1.889, 95% CI 1.267–2.815, P = 0.002; model 2, OR 1.693, 95% CI 1.048–2.736, P = 0.031) in both models when treated the tertiles of hemoglobin as a continuous variable (reference group: BG-EPVS > CS-EPVS for all comparisons) (Table 3).

**Table 3:** Multinomial logistic regression of associations between hemoglobin and EPVS degree predominance patterns.

	Model 1	Model 2	Model 1	Model 2
**Hemoglobin**				
Tertile 1	Ref	Ref	Ref	Ref
Tertile 2	2.154 (1.104–4.201)	2.463 (1.195–5.075)	1.441 (0.680–3.057)	1.658 (0.727–3.781)
Tertile 3	2.808 (1.329–5.934)	2.625 (1.102–6.251)	3.378 (1.529–7.466)	2.576 (1.004–6.608)
Hemoglobin (tertile 1–tertile 3)	1.690 (1.170–2.441)	1.679 (1.085–2.598)	1.889 (1.267–2.815)	1.693 (1.048–2.736)

The base outcome for all analyses is the BG-EPVS > CS-EPVS pattern.

Model 1: unadjusted model.

Model 2: adjusted for age and sex.

*BG* basal ganglia, *CS* centrum semiovale, *EPVS* enlarged perivascular spaces, *OR* odds ratio, *CI* confidence interval.

## Discussion

In this study, we investigated the association between hemoglobin level and the severity of EPVS in the Chinese population. The results showed an increase trend in the prevalence of high degree of CS-EPVS with increasing tertiles (tertile 1–tertile 3) of hemoglobin, while no significant trend was found in the prevalence of BG-EPVS. After adjusted for potential confounding variables, elevated hemoglobin was independently associated with high degree of CS-EPVS. Similar results were found as to the predominant EPVS topography that elevated hemoglobin was associated with higher odds of CS-EPVS predominance pattern. Our findings support that the topography of EPVS is characteristic: elevated hemoglobin was related to the severity of CS-EPVS but not BG-EPVS. To our knowledge, the association between elevated hemoglobin and the severity of CS-EPVS has not been reported before.

Recently, EPVS have been considered as a feature of SVD, similar to lacunes, WMH and CMB, in STRIVE recommendations^22^, but the relationship between EPVS and underlying small vessel pathologies is still under active investigation. Several studies have demonstrated that EPVS in different locations might have different pathogenesis^7,14^. In our study, BG-EPVS but not CS-EPVS were associated with hypertension, lacunes and WMH. These features have been demonstrated related to hypertensive arteriopathy, indicating that BG-EPVS might be a feature of hypertensive SVD. This was consistent with previous reports on the mechanisms of EPVS in different locations^7,13,14,17^. Hypertensive arteriopathy mainly affects the terminal perforating arteries of the brain^24^ and leads to thickened arteriolar wall, endothelia cell dysfunction and arterial stiffness^25,26^. Increased arteriolar pulsatility and restricted vasodilation then impair normal perivascular cerebrospinal fluid flow, resulting in stagnation of cerebrospinal fluid in perivascular spaces and further contributing to the development of BG-EPVS^27,28^.

By contrast, CS-EPVS have been shown to be associated with CAA, one of the protein elimination failure angiopathies that mainly derives from deposition of β-amyloid (Aβ) in the wall of small cortical and leptomeningeal arteries^29^. Previous rodents’ studies injected fluorescent soluble tracers into the corpus striatum and then examined the brains by confocal microscopy. They found that perivascular spaces of capillaries and arteries might act as drainage pathways of fluid and solutes out of the brain^30^. The link between CS-EPVS and CAA may specifically reflect ISF drainage dysfunction along the perivascular spaces caused by impeded elimination of metabolites (including Aβ and other proteins) from cortical and leptomeningeal arteries, which is a crucial and probably early event in the pathogenesis of CAA^31,32^. Previous study suggested that hemoglobin, a major Aβ-binding protein in cerebral circulation, was associated with the severity of CAA and might participated in the pathophysiology of CAA^20^. In our study, elevated hemoglobin was independently associated with high degree of CS-EPVS. Similar results were found as to the predominant EPVS topography that hemoglobin was associated with higher odds of CS-EPVS predominance pattern. Since no study have yet investigated the relationship between hemoglobin level and the presence or severity of EPVS, we cannot compare our results with those of other studies. We acknowledge that the association between elevated hemoglobin and the severity of CS-EPVS still needs further validation in larger, more varied cohorts.

Although elucidation of the mechanism responsible for the relationship between elevated hemoglobin and CS-EPVS will require further examination in a properly designed study, the elevated hemoglobin level could reflect the level of oxygen consumption and might associated with polycythemia and increased blood viscosity^33^. Coull et al.^34^ found severe blood hyperviscosity in patients with known vascular risk factors, suggesting that blood hyperviscosity may precede cerebrovascular disease symptoms. Previous studies also demonstrated that blood hyperviscosity is a risk factor for the development of multiple lacunes, another main feature of SVD^35,36^. It might be one of the reasons that linking hemoglobin and the development of EPVS. Besides, EPVS have been suggested as a maker of early blood–brain barrier (BBB) dysfunction^37^. Impairment of normal structures in the vascular smooth muscle and tunica adventitia, together with the dilation of vascular lumen, result in increased permeability of vascular endothelium in arterioles. Thus, impairing the regulatory function of the BBB and causing substances that normally stay in the blood to enter into the extravascular tissues^37,38^. These substances including blood components such as red cells were extravasated into the perivascular spaces, leading to enlargement of perivascular spaces and further aggravating the damage to small vessel wall^38^. High hemoglobin level as well as the increased blood viscosity will further aggravate the damage to BBB and extravasation of the blood components, and ultimately leads to development of EPVS.

What’s more, previous study using high-definition MRI has demonstrated the presence of hemoglobin and its products (haemosiderin) in the vessel wall of small deep perforating arteriole in patients with SVD^39^. Since hemoglobin was found to promote Aβ oligomer formation and colocalize with amyloid deposits, it is suggested that hemoglobin derived from cerebral circulation, may aggravate the obstruction of perivascular ISF drainage pathway and further contribute to the pathogenesis of EPVS^20^. Besides, accumulation of Aβ in perivascular spaces might activate the inflammation response and contribute to downstream damage to vascular endothelium and vessel wall, inducing the deterioration of BBB dysfunction^40,41^. This is in line with recent studies which suggesting that EPVS in the CS, but not in the BG, may be specifically attributed to systemic inflammation^42^.

Strengths of our study include the relatively large sample size, the systematic evaluation of MRI scans by trained rater using validated scales for a range of SVD imaging markers, and the use of prespecified EPVS cutoffs and patterns. While our study provides important information regarding the role of elevated hemoglobin in EPVS, we still need to take cautious when interpreting our data. First, our study was limited to a single-center and single-ethnicity sample with acute ischemic stroke, the results may not represent the general population and other ethnic groups. Second, we excluded patients with serious conditions that unable to tolerate MRI examination or have difficulty in EPVS assessment. This selection bias would probably limit the generalizability of the results to all stroke patients. Besides, the sample size of high degree BG-EPVS group is relatively small (n = 45), which might influence the statistical validity in multivariable analyses. Thus, the association between hemoglobin level and the severity of EPVS still needs further validation in larger, more varied cohorts. Third, we acknowledge that the EPVS visual rating scale used in our study, which, although validated, is not truly quantitative and may be affected by observer variability. However, the distinction between CS-EPVS vs BG-EPVS and the predefined cutoff used was sensitive enough to capture the topographic differences and independent associations consistently in different models. Fourth, this retrospective analysis of clinical and laboratory data does not allow for more detailed assumptions on the potential causality. Future prospective trials involving larger cohorts are needed to confirm the causal relationship between elevated hemoglobin and severity of CS-EPVS.

In conclusion, the present study has shown that elevated hemoglobin was independently associated with high degree of CS-EPVS and higher odds of CS-EPVS predominance pattern. Our findings support that the topography of EPVS is characteristic: hemoglobin was associated with the severity of CS-EPVS but not BG-EPVS, suggesting that EPVS in BG and CS might have different pathogenesis. However, elucidation of the mechanism responsible for the relationship between high hemoglobin level and CS-EPVS will require further examination in a properly designed study.

## Supplementary Information

Supplementary Information.

## Data Availability

The data that support the findings of this study are available from the corresponding author, upon reasonable request. The data are not publicly available as they contain information that could compromise the privacy of research participants.
