# Supplementary material for: Elevated hemoglobin is independently associated with enlarged perivascular spaces in the central semiovale
Source: Sci Rep. 2021 Feb 2;11:2820. doi: 10.1038/s41598-021-82327-9 (PMC7854720; doi:10.1038/s41598-021-82327-9)
Supplement: Supplementary file 1 — Supplementary Information. [file 41598_2021_82327_MOESM1_ESM.pdf]

Elevated Hemoglobin is Independently Associated with Enlarged Perivascular Spaces in the Central Semiovale

Yingchao Huo[1], Siyuan Huang[1], Rui Li[2], Xue Gong[1], Wenyu Zhang[1,3], Rongrong Zhang[1], Xinyue Qin[1*]

[1]Department of Neurology, The First Affiliated Hospital of Chongqing Medical University, Chongqing, 400016, P.R. China

[2]Department of Neurology, The First Affiliated Hospital of USTC, Division of Life Sciences and Medicine, University of Science and Technology of China, Hefei, Anhui, 230001, P.R. China

[3]Department of Neurology, Panzhihua Municipal Central Hospital, Panzhihua, Sichuan, 617000, P.R. China

Correspondence: Prof. Xinyue Qin, Department of Neurology, The First Affiliated Hospital of Chongqing Medical University, Chongqing, 400016, P.R. China; Email: qinxy2019@sina.com; Tel: +86 23 89012478; Fax: +86 23 89012478.

Methods

Definitions of lacunes, periventricular white matter hyperintensities (PVWMH) and deep white matter hyperintensities (DWMH):

White matter hyperintensities (WMH) were diagnosed and scored by the revised version of the visual scale of Fazekas and colleagues[1]. In the Fazekas rating scale, WMH are divided into periventricular white matter hyperintensities (PVWMH) and deep white matter hyperintensities (DWMH) according to anatomic location. PVWMH are scored as follows: none (0, no lesion); mild (1, caps or a pencil-thin lining); moderate (2, smooth halo); severe (3, irregular lesions extending into the deep white matter). DWMH are scored as follows: none (0, no lesion); mild (1, punctuate foci); moderate (2, beginning confluent foci); severe (3, large confluent lesions). Lacunes were defined as rounded or ovoid lesions, > 3 mm and < 20 mm diameter, in the basal ganglia, internal capsule, centrum semiovale, or brainstem, of CSF signal intensity on T2 and FLAIR, generally with a hyperintense rim on FLAIR and no increased signal on DWI[2].

References

1.Fazekas, F., Chawluk, J. B., Alavi, A., Hurtig, H. I., Zimmerman, R.A. MR signal abnormalities at 1.5 T in Alzheimer's dementia and normal aging. *AJR Am J Roentgenol* **149**, 351-356 (1987).
2.Wardlaw, J. M. *et al.* Neuroimaging standards for research into small vessel disease and its contribution to ageing and neurodegeneration. *Lancet Neurol* **12**, 822-838 (2013).

Table S1. Association between enlarged perivascular spaces and features of small vessel disease.

	Degree of CS-EPVS			Degree of BG-EPVS		
	Low (n=307)	High (n=94)	*P* Value	Low (n=356)	High (n=45)	*P* Value
Lacune			0.339			0.003
0	169(55.0)	57(60.6)		210(59.0)	16(35.6)	
≥1	138(45.0)	37(39.4)		146(41.0)	29(64.4)	
PVWMH			0.690			0.002
None	16(5.2)	4(4.3)		20(5.6)	0(0)	
Mild	135(44.0)	43(45.7)		167(46.9)	11(24.4)	
Moderate	106(34.5)	36(38.3)		121(34.0)	21(46.7)	
Severe	50(16.3)	11(11.7)		48(13.5)	13(28.9)	
DWMH			0.448			0.005
None	77(25.1)	23(24.5)		96(27.0)	4(8.9)	
Mild	146(47.6)	50(53.2)		175(49.2)	21(46.7)	
Moderate	47(15.3)	15(16.0)		52(14.6)	10(22.2)	
Severe	37(12.1)	6(6.4)		33(9.3)	10(22.2)	

Abbreviations: EPVS, enlarged perivascular spaces; BG, basal ganglia; CS, centrum semiovale; PVWMH, periventricular white matter hyperintensities; DWMH, deep white matter hyperintensities.

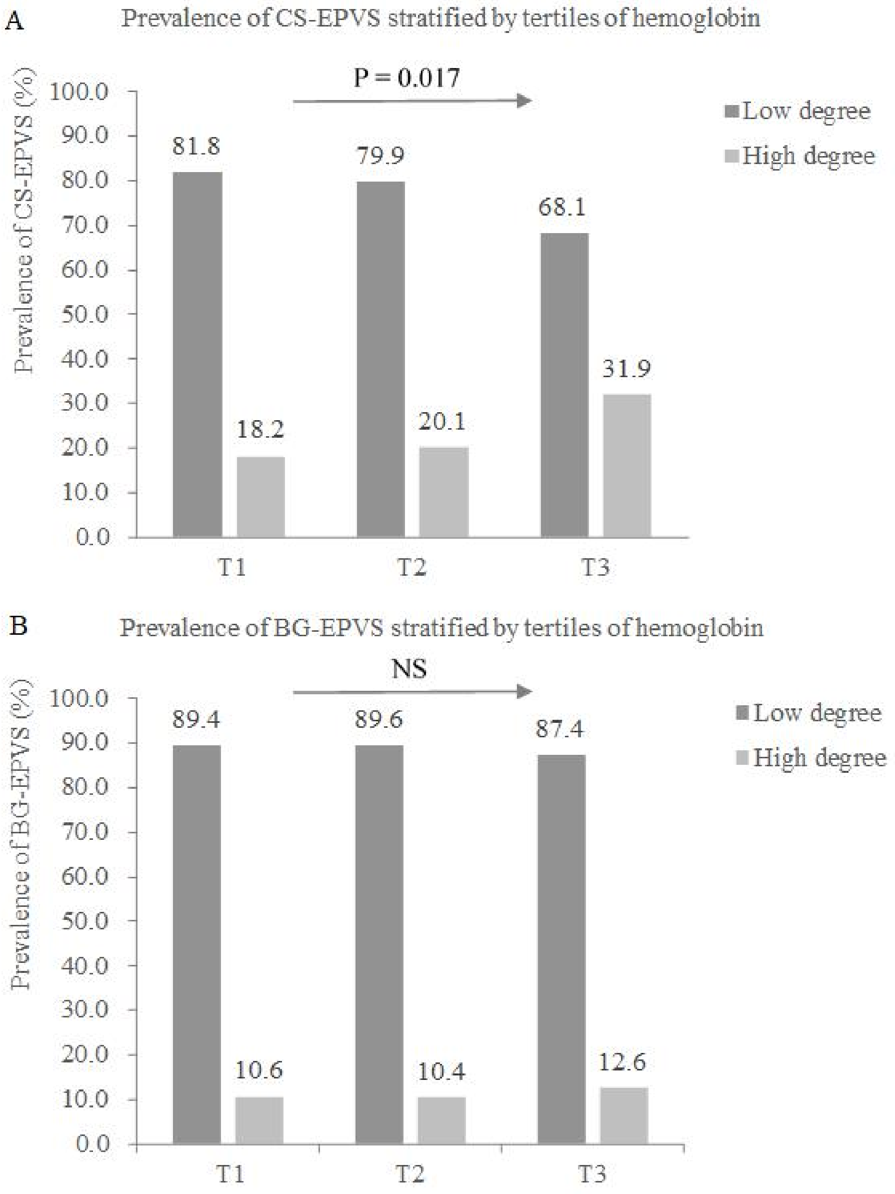

Figure S1. Prevalence of EPVS in CS and BG stratified by tertiles (Tertile 1 [T1]-Tertile 3 [T3]) of hemoglobin (A, B). The prevalence of low and high degree of CS-EPVS (A) and BG-EPVS (B) are showed above each column. With increasing tertiles of hemoglobin, the prevalence of high degree of CS-EPVS increased significantly (p=0.017), but the prevalence of high degree of BG-EPVS showed no differences among tertiles (p>0.05). NS, not significant. BG, basal ganglia; CS, centrum semiovale; EPVS, enlarged perivascular spaces.

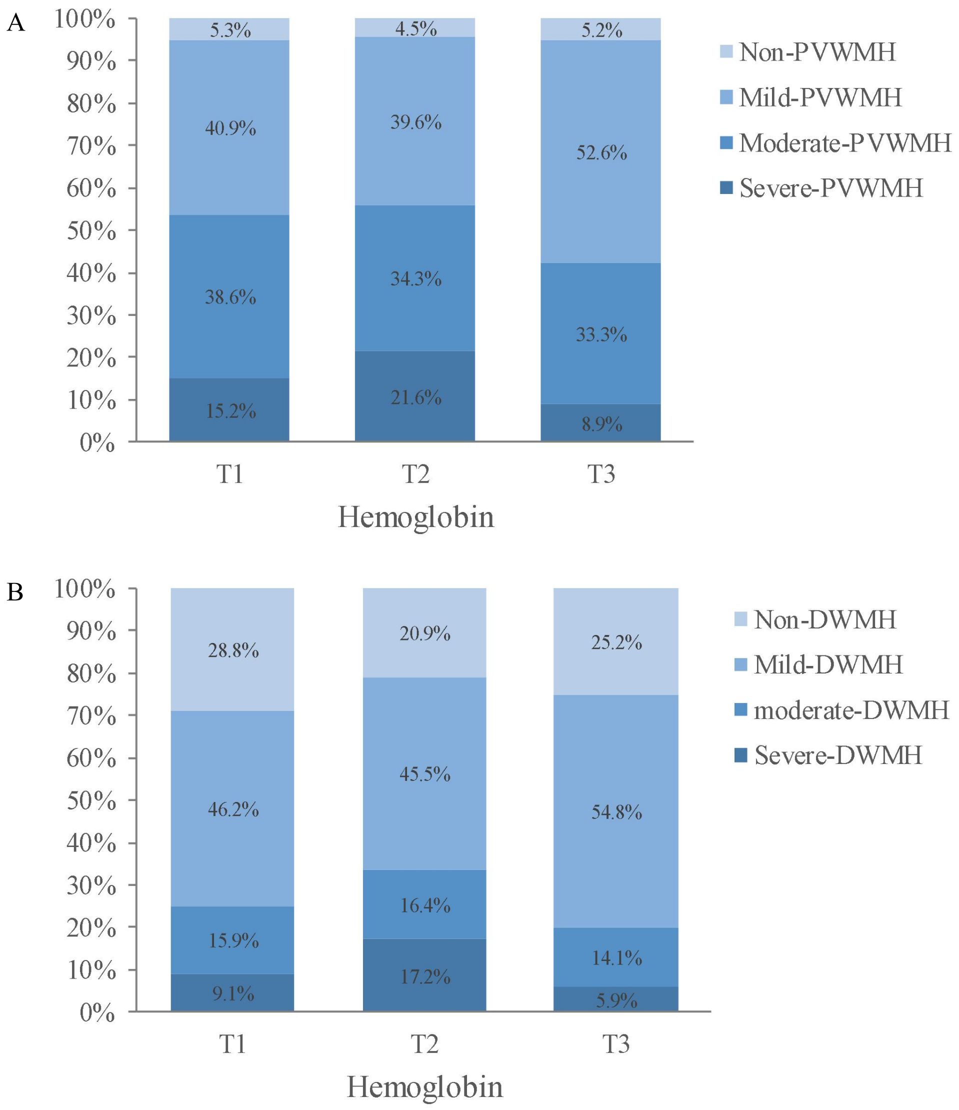

Figure S2. Distribution of PVWMH (A) and DWMH (B) severity according to tertiles (Tertile 1 [T1]-Tertile 3 [T3]) of hemoglobin. The percentage of PVWMH (A) and DWMH (B) severity is shown in each cell. The distribution of PVWMH and DWMH severity showed no significant differences between tertiles (T1-T3) of hemoglobin (ptrend=0.088, A; ptrend=0.066, B). PVWMH, periventricular white matter hyperintensities; DWMH, deep white matter hyperintensities.
